# Universal Access to Healthcare: The Case of South Africa in the Comparative Global Context of the Late Anthropocene Era

**DOI:** 10.34172/ijhpm.2020.28

**Published:** 2020-03-01

**Authors:** Solomon Benatar, Stephen Gill

**Affiliations:** ^1^University of Cape Town, Cape Town, South Africa.; ^2^Dalla Lana School of Pubic Health, University of Toronto, Toronto, ON, Canada.; ^3^Political Science, Communications and Culture, York University, Toronto, ON, Canada.

**Keywords:** Universal Healthcare Access, South Africa, Anthropocene, Social Innovation

## Abstract

Much current global debate – as well as a great deal of political rhetoric – about global health and healthcare is characterised by a renewed emphasis on the goal of universal access throughout the world. While this goal has been achieved to varying extents in the United Kingdom, Canada and many countries in Europe, even within those countries where national health systems have long been in place, the pervasive shift in emphasis from health as a social value to health as a commodity within a capitalist market civilization is eroding the commitment to equitable access to healthcare. Against this background the challenge is much greater in low- and middle-income countries that lag behind – especially if aspirations to universal access go beyond primary care. The challenges of achieving greater equity in access to health and in health outcomes, in a middle-income country like South Africa, illustrate the magnitude of the tensions and gaps that need to be traversed, given the vast differences between healthcare provided in the private and public sectors. Understandably the concept of National Health Insurance (NHI) in South Africa has widespread support. The strategies for how a successful and effective NHI could be implemented, over what time-frame and what it covers are, however, very controversial issues. What tends to be ignored is that sustainable improvement in health in South Africa, and elsewhere, is not determined merely by medical care but more especially by social structures intimately linked to deeply entrenched local and global social, economic and political forces and inequalities. While seldom openly addressed, some of these forces are explicated in this article to supplement views elsewhere, although most have elided emphasis on the pervasive effects of the global political economy on the provisioning and practising of health and healthcare everywhere on our planet.

## Introduction

In the early 21st century the condition of the world embraces a complex set of interlinked and pervasive structural crises. These include wide and widening disparities in health and human rights,^1^ emergence and spread of new infectious diseases due to close interactions between animals and humans in severely deprived ecological niches (illustrated by the newly emerging Corona Virus that reminds us of the potential for future pandemics that could rapidly kill millions of people world-wide),^2^ increasing resistance to drugs for treating tuberculosis and many other infections,^3^ ongoing human belligerence in many countries fuelled by weapons purchased from the (so-called) developed world,^4,5^ politically constructed clashes of civilizations along with persistent racism, discrimination, and marginalization,^6,7^ and not least intensification of both progressive and reactionary political anger.^8^ Together these crises and crisis tendencies form part of what has been termed a “global organic crisis,” involving unsustainable trajectories of accelerating energy intensive, market-driven economic development that pose threats to the health and well-being of people, the planet and its biosphere. Its most adverse components involve the combination of multi-dimensional socio-economic inequalities, environmental degradation^9^ and climate change,^10^ all driven by patterns of wasteful and often frivolous consumerism within a political order and market civilization where economic growth and profit dominate over public interests.^11,12^ This combination, more recently labelled a ‘planetary crisis,’^13^ contributes to escalating humanitarian distress, internal displacements of millions of people and mass migrations of international refugees escaping from atrocious living conditions and conflict.^14^

It is in this global context that we must consider the question of universal access to healthcare – a goal widely stated as a global priority.^15^ However, the realities of local and global conditions, configured by the dynamics of an ecologically unsustainable global political economy, have undermined pursuit of this goal.^16^ The notion of universal access has thus become more rhetorical than real, and new principles and practices are needed to make it a more achievable goal. To understand why this goal has proven elusive we explore the case of a society which has sought to emancipate its collective future by ridding itself of the curse of apartheid:

## The South African Case

The global organic crisis is mirrored locally by: (*i*) the trajectory of changes in health and healthcare in the 26 years since South Africa underwent a peaceful transition from apartheid to a fledgling constitutional democracy,^17-21^ (*ii*) environmental crises,^22^ (*iii*) a heavy burden of infectious diseases,^23^ (*iv*) widespread corruption within a severely dysfunctional economic system,^24^ and (*v*) political and social conflict, and xenophobia associated with influx of migrants.^25^

### Wealth Inequality

Currently the top 10% of South Africans earn 58% of the total annual national income, whereas the bottom 70% combined earn a mere 17% while 45% of the population continue to live on approximately $2 per day. Unemployment rates are high (29% overall and 50% amongst younger people) and relative poverty has worsened (Gini coefficient increased from 0.6 in 1995 to almost 0.7 in 2009).

### Health Inequalities

South Africa (0.7% of world population) bears a heavy burden of infectious diseases: eg, 17% of the global burden of human immunodeficiency virus (HIV) infection and one of the worst tuberculosis epidemics in the world. Driven by the spread of HIV infection, the incidence of tuberculosis increased from 300 per 100 000 people in the early 1990s to more than 600 per 100 000 in the early 2000s and to more than 950 per 100 000 in 2012, with multidrug-resistant tuberculosis accounting for about 2% of the disease burden and consuming about 30% of the tuberculosis budget. Life expectancy at birth is much lower than in other countries with similar economic levels.

### Healthcare in Public and Private Sectors

Annual per capita expenditure on health of about $500 (with about 50% of total expenditure in each of the private and public sectors) ranges from about $1500 in the private sector (for 17% of South Africans who have private health insurance to cover care provided by some 70% of the doctors in the country) to about $150 in the public sector (for approximately 83% of the population who are uninsured and served by 30% of the country’s doctors), with many public sector hospitals and services in a state of crisis, as a result of neglect, underfunding and mis-management. Disease profiles also differ widely. Communicable diseases dominate in the public sector where there is also limited access to modern surgery and other sophisticated treatments for chronic non-communicable diseases. A reverse pattern characterizes the private sector.

All the above disparities in wealth, health and disease burden and their impact in South Africa (a microcosm of global conditions) hamper the task of building on the country’s social strengths and wealth to advance the processes and outcomes outlined in South Africa’s new constitution and the widespread hope for a better society. Many years ago the point was made that if the political transition was not accompanied by social progress towards narrowing disparities and improving the lives of the previously oppressed, the fight over political power would be transformed into a war over resources.^19^ The question was also raised whether progress in the new South Africa could be sustained if other countries failed to shift their paradigms towards narrowing their own disparities^26^ in the wider context of a form of global apartheid also in need of rectification by reducing exploitation of others.^27^

### Universal Access to healthcare and Prospects for NHI in South Africa

Universal access to healthcare has become a global priority, and working toward the goal of National Health Insurance (NHI) in South Africa, to provide more equitable access to high quality individual health services, has reemerged as a popular notion.^28^ While health economists in South Africa suggest that it is feasible to raise the additional required funding for NHI, expectations that equity in healthcare delivery could be achieved at levels close to current private-sector levels are economically and practically unrealistic,^29^ and could also provide yet another means of ‘state capture’ as exemplified in other state-owned enterprises.^30^

Regrettably, the World Health Organization (WHO) and others, who are actively promoting universal access, are not defining achievable *levels* of universal access. With 70% of the world’s population living on less than US$10 a day and 50% on less than US$4 a day, there is an urgent need for discussion and debate about what could be achieved within specified time frames, and the specific strategies required — through more regulated local and global political economies and upgraded notions of poverty alleviation to reduce dehumanizing disparities.^31^

## Health and Wealth in an Unequal World

At the global level, the top 10% earn 52% of the total annual income, whereas the bottom 70% combined earn a mere 19%: the global Gini coefficient has increased during recent decades.^32^ When extreme poverty affects a large proportion of the population, health is predominantly affected by a lack of access to the basic requirements for life — clean water, adequate nutrition, effective sanitation, reasonable housing conditions, access to vaccinations, education, and the childhood and adolescent nurturing that, with the availability of jobs, set the scene for improved health and longevity. Life expectancy at birth globally ranges from less than 50 to over 80 years.

The wealthiest 20%-30% of the world’s people seem to have endless expectations and live as if their privileged lives will improve indefinitely, and limitless attempts to prolong individual life are prioritized over addressing the basic social forces that powerfully determine the health of populations generally.^33^

Whilst capitalist practices imply that ongoing economic growth, allied to scientific/technological ingenuity will improve health, global planetary limits are being exceeded.^34^ In the Anthropocene era social and economic activities, driven by the ‘civilizational’ forces and patterns, the inequalities, conflicts and crises noted above have escalating environmental impacts with serious asymmetrical implications for health locally and globally. Notably the world’s richest 10% produce 50% of global carbon emissions while the poorest 50% contribute to just 10% of emissions.^35^ These consumption patterns are principally driven by a small number of massive carbon capitalist private corporations such as Exxon and their state-owned counterparts like Saudi Aramco, providing the carbon lifeblood of an energy intensive, consumerist and ecologically myopic market civilization. Consequent threats to the health and well-being of all globally have been vividly illustrated by recent aberrant weather trends and consequent rising death rates,^36^ most especially in the ‘global south,’ with predictions of much worse ahead.^37,38^

The central and critical role of the global political economy within the global/planetary health scenario, has not yet been adequately acknowledged in an era of high technology medicine where hope for progress is increasingly focused on genomics, personalized medicine, big data and artificial intelligence.^39^ Growing global political instability and dislocations, emerging from the widening gulf between the world’s ‘haves’ and ‘have-nots,’ now call for immediate understanding of health as a complex political, social, economic and ultimately eco-centric notion.^40,41^

## From the Local to the Global: A Fatal Diagnosis

In attempting to understand responses to these local and global situations it may be helpful to draw an analogy between the response to a fatal diagnosis in an individual person and the response to a fatal diagnosis for life (as we know it) on our planet at a late stage in the Anthropocene era. Seventy percent of all deaths in the world are due to non-communicable diseases^42^ and these develop slowly, with symptoms and signs of disease only manifesting late in their progress. For example, when a coronary artery becomes progressively narrowed over many decades, symptoms generally only arise when the artery is 70%-90% occluded. Another example is when, after many years of smoking, a single cell in the lung becomes malignant, it takes (for some variants of lung cancer) approximately 100 days to become 2 cells and another 100 days to become 4 cells. It takes 30 such doublings, at a constant rate over 9 years, for the cancer to reach 1 cm in diameter (earliest visibility on a chest radiograph). At 40 doublings the cancer is 10 cm in diameter and death will have resulted (death can also occur much earlier if the cancer has spread).^43^ When the diagnosis of these potentially fatal diseases is made, patients are often surprised, as they do not feel so very ill and yet the prognosis is poor unless effective medical treatment is available and can be instituted. According to the Kubler-Ross analysis the sequence of grief responses to a fatal diagnosis is denial, anger, depression, bargaining, and then acceptance.^44^

Moving from the individual person to the planet we consider the potentially fatal diagnosis of a ‘chronic disease’ – ecological entropy resulting from decades of exponential energy use with slow, but relentless loss of nature’s ability to compensate for ongoing environmental damage.^45^ The symptoms, physical manifestations and explanations for the pre-terminal stage are wide-ranging and, as with diseases in individuals, only manifest late in the deteriorating process.

As with bad news for individuals, denial of the implications of many threats to health and future lives globally has been the first ‘public’ response to this diagnosis of a potentially fatal disease of our planet – and by implication of all human life.^46^ Ongoing pursuit of continuous economic growth and myopic expectations for the future are symptomatic of denial about the dying of our planet.^47^ Climate concern is at last widely acknowledged,^48,49^ and a 2050 doomsday scenario has been envisaged.^46,50^

Associated recent political trends leading away from cosmopolitan internationalism towards nationalism and reactionary forms of internationalism, include the rise of right-wing attitudes and political parties, intensified armament strategies and protectionist trade rules. These all reflect a trend towards a transactional view of politics, allied to ‘fortress mentalities’ to ensure the short-term security of capital for the powerful. These trends are in keeping with the Kubler-Ross stages of anger, depression and bargaining. Movement beyond all these stages towards rapid acceptance is the prerequisite for appropriate action.^51^

Whether or not we are able to take action to redress these morbid symptoms will depend on understanding the processes through which we have reached our current impasse. Given blind faith in the structure and functioning of the global political economy by those who have gained most from this, it would be a tough call to expect dramatic change. However, it is not beyond human ingenuity to find ways of reducing conflict and ameliorating climate change. This will require, inter alia, new conceptions of ourselves conducive to moderation of exorbitant expectations and entitlements currently taken glibly as ‘normal,’ reduction in exponential use of energy together with redistribution through progressive taxation, reduction in tax havens^52^ and efforts to reduce the pervasive corruption that threatens democracy world-wide.^53^ Some wealthy countries are more resilient than others and will probably survive these ecological and other threats for longer than others. Yet none can escape, and such threats therefore cannot and should not be ignored.

## Making Progress: New Practices, New Imaginaries

A pre-requisite for embarking on 21st century shifts in thinking and action that could sustain and expand equitable access to healthcare and promote significant improvement in global health, entails accepting the discomfort of acknowledging the social and societal determinants of health,^54^ and how the structure of the local and global political economy, deeply embedded and distorted power relations,cultural complexities, pervasive corruption and poor global leadership,^55^ all perpetuate intractable and dehumanizing disparities.^13,31,56^

Acknowledgment of the severity and threat of social disruption in countries with profoundly inequitable access to healthcare, and globally in relation to planetary malaise,^57,58^ could encourage constructive collective action for transforming the status quo. Promotion and improvement of health (locally and globally) is inherently a political endeavour dependent on a sophisticated understanding of power relations/dynamics,^59^ and the use of sociological^60,[1]^ and moral imaginations^61,[2]^ to shift towards a global frame of mind favouring innovative social change. New paradigms of citizenship,^62^ encouraged and driven by an expanded ethical discourse^63^ and deeper understanding of long-term self- and collective-interest, could hopefully reinforce insight into the now critical interconnectedness and mutual interdependency of humans on each other and nature.^12^

New metaphors for living that could helppromote such new frameworks of thinking and behaving,^64^ to achieve well-being, health and security for a greater proportion of the world’s population through the 21st century and beyond.^65^ include *developing sustainability,*^66^ *cultivating the future*,^67^ considering *responsibilities as well as rights* and *doing better with less.*^68^

The long-recognised need for visionary and credible leadership in global health has now become crucial.^69^ Our intentions may be admirable, and we have the intellect and the material resources to achieve this goal, but the big question is whether we have the vision and political commitment to transform a fraudulent and corrupt global political economy.^51,70^ Hope for such *innovative social progress* lies in the abundance of human ingenuity as reflected in *innovative science*.^71^ Scientific and technical advances and moral aspirations must now be accompanied by the socio-political research and ethically appropriate actions required to reverse the shortcomings of our complex socially constructed world with its increasingly fragile economic and ecological systems.^72^

Ignoring these challenges and failing to act with vision and wisdom confines us within a cycle of ongoing wasteful consumption patterns, persisting wide disparities, violence and destruction within nations and globally. The proliferation of books about the Anthropocene (see https://www.goodreads.com/shelf/show/anthropocene),the failures of unrestrained capitalism^73^ and instructive recent documentaries reveal dire implications that cannot be ignored.^74-76^

## Conclusions

The current rhetoric about universal access to healthcare both in South Africa and globally is undermined by hypocrisy, failure to define the extent of recommended universal access, denial of core causal factors in determining health and abject failure to address these. An expanded consciousness is needed to develop a sustainable eco-centric world-view and to innovate actions and policies that could begin to achieve more equitable access to healthcare while ameliorating already evident social and environmental tragedies in local and global contexts. While it may be doubtful whether we are capable of adapting to a paradigm appropriate for innovatively addressing such 21st century challenges, we should at least attempt to do so through meaningful, cross-cultural, trans-disciplinary dialogue and research^77^ in pursuit of much needed, cosmopolitan political, moral and humanitarian leadership, in conjunction with activist social movements.^12,51,78,79^

## Ethical issues

Not applicable.

## Competing interests

We have no conflicts of interest. The article has not been published or submitted to any other journal for publication consideration.

## Authors’ contributions

SRB conceived the article and wrote the initial drafts. Many of the ideas were drawn from collaborative work by both authors over many years. Both authors contributed to the final manuscript and its revision and approved it.

## Authors’ affiliations

^1^University of Cape Town, Cape Town, South Africa. ^2^Dalla Lana School of Pubic Health, University of Toronto, Toronto, ON, Canada. ^3^Political Science, Communications and Culture, York University, Toronto, ON, Canada.

## Endnotes


[1] Enables understanding of the larger historical influences on the inner and external lives of individuals and allows recognition of how daily experience often generates false consciousness of our social positions.

[2] The ability of individuals and communities to empathize and connect interdependently with others.

